# Identification of apple genes Md-Exp7 and Md-PG1 alleles in advanced selections resistant to scab

**DOI:** 10.18699/VJGB-22-79

**Published:** 2022-11

**Authors:** I.I. Suprun, S.V. Tokmakov, E.A. Al-Nakib, E.V. Lobodina

**Affiliations:** North Caucasian Federal Scientific Center of Horticulture, Viticulture, Wine-Making, the Functional Scientific Center of “Breeding and Nursery”, Krasnodar, Russia; North Caucasian Federal Scientific Center of Horticulture, Viticulture, Wine-Making, the Functional Scientific Center of “Breeding and Nursery”, Krasnodar, Russia; North Caucasian Federal Scientific Center of Horticulture, Viticulture, Wine-Making, the Functional Scientific Center of “Breeding and Nursery”, Krasnodar, Russia; North Caucasian Federal Scientific Center of Horticulture, Viticulture, Wine-Making, the Functional Scientific Center of “Breeding and Nursery”, Krasnodar, Russia

**Keywords:** apple, breeding, marker-assisted selection, fruit quality, scab resistance, Md-Exp7, Md-PG1, Md-ACS1, Rvi6, complex donors, gene pyramiding, яблоня, селекция, маркер-опосредованный отбор, качество плодов, устойчивость к парше, Md-Exp7, Md-PG1, Md-ACS1, Rvi6, комплексные доноры, пирамидирование генов

## Abstract

The creation of apple varieties with a high level of f lesh f irmness and long shelf life is one of the important goals in breeding. Among the genes controlling these traits, the role of the endogenous ethylene biosynthesis control gene, Md-ACS1, the expansin gene, Md-Exp7, and the polygalacturonase gene, Md-PG1, has been established. The use of DNA marker analysis to solve problems in breeding for fruit quality traits allows one not only to track several target genes simultaneously, but also to cull plants with undesirable alleles at the early stages of development. In order to select complex donors of breeding traits, molecular genetic identif ication of the genes that determine the quality traits of apple fruits Md-Exp7 and Md-PG1 was performed in 256 breeding selections carrying the scab resistance gene Rvi6 and valuable allelic variants of the Md-ACS-1 gene, which determines the endogenous synthesis of ethylene in fruits: 90 samples with the Md-ACS1 allele (2/2) and 166 samples with Md-ACS1 (1/2). As a result of the study, an allelic combination for the Md-Exp7 and Md-PG1 genes was established. Analysis of the parental cultivars (Renet Simirenko, Modi, Smeralda, Renoir, Fulzhion and Granny Smith) used to obtain hybrid selections revealed three alleles 198, 202, 214 bp according to the DNA marker of the Md-Exp7 gene. The SSR marker for the Md-PG1 gene amplif ied three alleles (289, 292, 298 bp) on the abovementioned cultivars. Within the 256 breeding selections samples that have the most priority for breeding alleles of the desired genes in combination with the Rvi6 gene and/or with selection-priority allelic variants of the Md-ACS-1 gene were identif ied. Of the most valuable for breeding, 46 accessions carrying the combi nation Md-Exp7 (202:202) + Md-ACS1 (2/2) were distinguished. Hybrids with alleles Md-PG1 (292:292) + Md- ACS1 (2/2) are also most valuable for use in breeding and as donors of selection-valuable alleles; 21 samples were identif ied. Accessions with a complex of breeding-valuable target alleles are valuable complex donors, as well as valuable breeding material for creating varieties with improved fruit quality characteristics and scab resistance.

## Abstract (ru)

Создание сортов яблони, обладающих высоким уровнем плотности мякоти и лежкоспособности плодов, является одной из важных задач в селекции. Среди генов, контролирующих данные признаки, установлена роль гена контроля биосинтеза эндогенного этилена Md-ACS1 и генов экспансина Md-Exp7 и полигалактуроназы Md-PG1. Применение ДНК-маркерного анализа для решения задач в селекции на признаки качества плодов позволяет не только отслеживать одновременно несколько целевых генов, но и проводить выбраковку растений с нежелательными аллелями, не дожидаясь вступления гибридов в плодоношение, благодаря чему можно ускорить процесс отбора селекционно ценных форм. В целях отбора доноров по комплексу селекционно приоритетных аллелей с использованием мультиплексной ПЦР была выполнена молекулярно-генетическая идентификация генов Md-Exp7 и Md-PG1 у 256 селекционных форм, которые содержат ген устойчивости к парше Rvi6 и аллельные варианты гена Md-ACS-1: 90 образцов – Md-ACS1 (2/2) и 166 образцов – Md-ACS1 (1/2). Анализ родительских сортов яблони (Ренет Симиренко, Моди, Смеральда, Ренуар, Фулжион и Грени Смит), использованных при получении гибридных форм, выявил три аллеля длиной 198, 202, 214 п. н. по ДНК-маркеру, разработанному для гена Md-Exp7. SSR-маркер для гена Md-PG1 амплифицировал три аллеля (289, 292, 298 п. н.) в геноме родительских сортов. Генотипирование гибридных форм яблони позволило обнаружить образцы, содержащие сочетание приоритетных аллелей генов Md-Exp7, Md-PG1 и Md-ACS-1. В качестве доноров ценных аллелей отобрано 46 образцов, несущих комбинацию Md-Exp7 (202:202) + Md-ACS1 (2/2). Среди изученных гибридов обнаружен 21 образец, содержащий аллели генов Md-PG1 (292:292) и Md-ACS1 (2/2). Образцы с различным сочетанием селекционно ценных аллелей генов Md-Exp7, Md-PG1, Md-ACS-1 и Rvi6 рекомендуются для создания сортов с высоким уровнем лежкоспособности плодов и устойчивостью к парше.

## Introduction

Some of the most important traits of fruit quality in an apple are the flesh firmness and long shelf life of fruits. These traits not only form consumer attractiveness, but also provide an increase in the economic efficiency of the apple fruits produc- tion industry by improving storability of the fruits and their transportability. In this regard, the creation of varieties that have a firm texture of the flesh and preserve it during storage is an important direction in breeding. The change in the structure of the fruit flesh during fruit ripening and storage is regulated by various physiological and biochemical processes, among which an important role belongs to the process of endogenous synthesis of ethylene, an increase in the intensity of which leads to softening of the flesh due to the activation of various enzymatic systems that affect the density of the cell wall (Ji, Wang, 2021

Among the genes controlling the biosynthesis of endoge nous ethylene, in the apple, the key role belongs to the Md-ACS1 and Md-ACO1 genes encoding the enzymes 1ami - nocyclopropane1carboxylate synthase (ACCsynthase 1) and 1aminocyclopropane1carboxylate oxidase (ACCoxi dase1), which sequentially, in a chain of reactions, convert Sadenosylmethionine to ethylene (Dong et al., 1991, 1992; Kende, 1993). These genes have been mapped, the effect of allelic variants of genes on the level of endogenous ethylene synthesis in fruits and, accordingly, on the storage quality of fruits, has been established, and effective DNA markers for identifying alleles have been developed (Sunako et al., 1999; Oraguzie et al., 2004; Costa et al., 2005). Using these markers, allelic combinations in the breeding material and collection samples of the apple tree were assessed in the world (Oraguzie et al., 2007; Zhu, Barritt, 2008; Nybom et al., 2012; Suprun, Tokmakov, 2013; Savel’ev et al., 2014b; Lyzhin, Savelyeva, 2020; Shamshin et al., 2020). The influence of the Md-ACS3a gene on the synthesis of endogenous ethylene in fruits was also revealed (Bai et al., 2012). However, the contribution of this gene to the formation of this trait is lower than that of Md-ACS1 (Dougherty et al., 2016).

Along with the abovementioned genes, the expansin gene – Md-Exp7 and the polygalacturonase gene – Md-PG1 play an important role in the control of physiological and biochemical processes associated with the formation of the flesh structure and the preservation of its density during storage in the apple tree. Expansin is a protein involved in the enzymatic rearrange- ment of cell walls by breaking noncovalent bonds between the hemicellulose matrix and cellulose microfibrils, which increases the susceptibility of this structural polymer to the action of other enzymes (Cosgrove, 2000). The activity of the ethylenedependent enzyme polygalacturonase contributes to the destruction of the structure of the cellular pectin polymer by biochemical catalysis of the hydrolytic cleavage of (1–4) galacturonan (Brummell, Harpster, 2001).

In genetic studies of the Md-Exp7 and Md-PG1 genes, mi- crosatellite markers cosegregating with them were identified. For the microsatellite marker MdExp7SSR of the Md-Exp7 gene, localized in the first linkage group, it was found that an increase in the size of the amplification product correlates with the level of fruit flesh softening during storage: for a frag- ment of 198 bp characterized by a lower level of softening, for 202 bp – medium and for 214 bp – the highest (Costa et al., 2008). H. Nybom (Nybom et al., 2012) made a prelimi- nary conclusion about a possibly more significant effect of the allele with the size of the amplification product at the Md Exp7SSR microsatellite locus of 202 bp in comparison with the 198 bp allele. The polygalacturonase gene – Md-PG1, mapped at a distance of 37 cM from the Md-ACO1 gene in linkage group 10, has a more pronounced contribution to the phenotypic variation in the change in flesh density during storage of fruits at temperatures close to room temperature, and not in refrigerators in the temperature range 2–4 °С (Costa et al., 2010). This is of great importance for commer- cial attractiveness of the fruits stored during transportation without compliance of the temperature regime, in temporary warehouses of shopping malls, and in logistics centers. The studies revealed a number of DNA markers closely linked to this gene (Costa et al., 2010), including the microsatellite marker MdPG1 10kd, which is the most informative (Longhi et al., 2013b). Analysis of allelic variants of this DNA marker showed that the presence of an allele with a fragment size of 298 bp is undesirable for breeding varieties with improved flesh density retention without special storage conditions. At the same time, the homozygous variant for the allele 298 bp is the least promising for use in breeding (Longhi et al., 2013a)

It is noteworthy that a high level of influence on the phe- notypic manifestation of the trait was found at temperatures close to room temperature not only for the Md-PG1 gene (Costa et al., 2010), but also for the Md-ACS1 gene. When comparing data on allelic variants of the Md-PG1, Md-ACS1 and Md- ACO1 genes and the level of fruit flesh density in 108 apple varieties at the stage of harvesting maturity and after 20 days of storage (at a temperature of 20–25 °С) after harvesting, a relationship was established between the allelic variants of the Md-PG1 and Md-ACS1 genes and the degree of reduction in fruit flesh density (Kwon et al., 2020). Using the DNA markers of the Md-PG1 and Md-Exp7 genes, a number of studies were carried out to identify their alleles, including cultivars and species specimens of the genus Malus (Costa et al., 2008; Longhi et al., 2013a, b; Nybom et al., 2013; Savel’ev et al., 2014a; Shamshin et al., 2018; Savelyeva, Lyzhin, 2019; Dolzhikova et al., 2020), for the purposes of breeding and a part of the study of the allelic diversity of these genes within the genus Malus. For the Md-PG1 and Md- ACS1 genes, allelespecific SNP markers were also deve loped and further integrated into the SNParray for MASselection – Interna- tional RosBREED SNP Consortium OpenArray v1.0, which allows for the total detection of alleles of 11 genes (Chagné et al., 2019).

Obviously, the presence of DNA markers for genes that determine such economically valuable traits as flesh density and preservation of its characteristics during storage makes it possible to increase the efficiency of the breeding process, as well as to conduct prebreeding work for more efficient selection of parental pairs for crossing. Especially relevant is the issue of using DNA markers for the analysis of the allelic composition of genes that determine quality traits in connection with the polygenic control of this trait and the dif- ferent contribution to the phenotypic manifestation of the trait depending on the combinations of alleles of different genes: Md-ACS1, Md-ACO1, Md-PG1 and Md-Exp7. An important advantage of using markerassisted selection is the ability to simultaneously track several genes that control not only one, but several traits, including resistance to pathogens.

As part of our previous work, using DNA marker analysis, we created a wide range of apple breeding selections carrying the Rvi6 scab resistance gene in combination with various alleles of the Md-ACS1 gene. Expansion of the set of priority genes, the alleles for which will be identified in the created breeding selections, will make it possible to select the most valuable material for breeding. In this regard, in the presented study, the task was to identify the alleles of the Md-PG1 and Md-Exp7 genes in apple samples carrying the Rvi6 gene and selectionvaluable variants of the Md-ACS1 gene alleles (1/2, 2/2) to create apple varieties that combine a complex of economically valuable traits.

## Materials and methods

The object of research was 256 apple selections obtained in six combinations of crossing: (1) Renet Simirenko/Modi (62 pcs); (2) Renet Simirenko/Smeralda (65 pcs); (3) Renet Simirenko/Renoir (33 pcs); (4) Renet Simirenko/Fujion (22 pcs); (5) Renoir/Granny Smith (9 pcs); (6) Modi/Granny Smith (65 pcs). The hybrids were obtained earlier as part of a markerassisted breeding program aimed at the development of apple scabresistant varieties with improved fruit quality characteristics. The presence of the scab resistance gene Rvi6 (Suprun et al., 2018), as well as breedingvaluable alleles of the Md-ACS1 gene, was previously determined by DNAmarker based analysis

For DNA extraction, a CTABbased method was used (Mur - ray, Thompson, 1980). Molecular genetic identification of the alleles of the Md-PG1 and Md-Exp7 genes was performed using microsatellite markers MdPGl 10kd and MdExp7SSR, respectively (Costa et al., 2008; Longhi et al., 2013b). The analysis was carried out by two markers simultaneously in one PCR reaction, which included: 20 ng of DNA, 1.5 mM dNTPs, 10 pM of each primer, 1 u. Taq polymerase and 2.5 mM 10×standard PCR buffer. PCR program: 94 °C – 150 s, 32 cycles: 60 °C – 45 s, 72 °C – 60 s, 94 °C – 30 s; 1 cycle 72 °C – 10 min. Electrophoresis of PCR products was carried out on an automatic genetic analyzer Nanofor 05. Analysis of the results was performed using the GeneMarker V3.0.1 program.

## Results

**Fig. 1.**
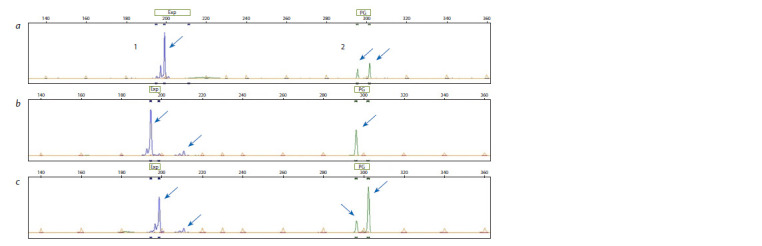
Multiplex fragment analysis of amplification products for DNA markers of the Md-Exp7 (1) and Md-PG1 (2) genes. The electropherogram shows examples of the results of the analysis of a sample homozygous for Md-Exp7SSR and heterozygous for Md-PG110kd (a); hetero- zygous for Md-Exp7SSR and homozygous for Md-PG110kd (b) and simultaneously heterozygous for two target loci (c).

The absence of overlapping in the size ranges of the ampli- fied fragments by the used DNA markers (198–214 bp for the Md Exp7SSR marker and 289–302 bp for the MdPG1 10kd marker) made it possible to apply multiplex identification (Fig. 1)

Analysis of the parental cultivars used to obtain apple selections revealed three fragments, 198, 202, 214 bp in size by the marker of the Md-Exp7 gene, while fragments of 289, 292, 298 bp in length were identified by the SSR marker of the Md-PG1 gene (Table 1).

**Table 1.**
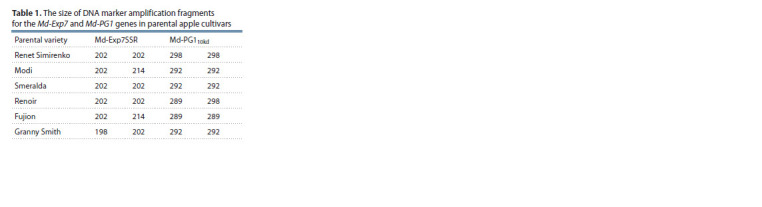
The size of DNA marker amplification fragments for the Md-Exp7 and Md-PG1 genes in parental apple cultivars

DNA markerbased analysis of hybrid plants revealed vari - ous combinations of alleles. Taking into account the fact that for the MdExp7SSR marker in the parental cultivars the allele with the size of the amplified fragment of 202 bp was most common (represented in all varieties, wherein in the varieties Renet Simirenko, Smeralda and Renoir in the homozygous state), its presence was detected in all hybrid samples, with the exception of 21 hybrids from combination No. 6 (Modi/ Granny Smith), carrying the allelic combination 198:214. At the same time, the allele 202 bp in the homozygote was pres- ent in 113 samples. Allelic combinations 198:202 and 202:214 were found in seven and 115 hybrid plants, respectively. Iden- tification of the alleles of the Md-PG1 gene marker revealed that the most common was the allele with a product size of 292 bp, while in 65 samples it was found in the homozygote. Along with the allelic variant 292:292, allelic combinations 289:292 were identified (7 samples); 292:298 (129 samples); 289:298 (38 samples) and 298:298 (17 samples).

## Discussion

Molecular genetic analysis of parental cultivars based on DNA markers of the Md-Exp7 and Md-PG1 genes made it possible to identify allelic combinations for a number of cultivars for the first time, as well as confirm the already available scien- tific information for the Granny Smith and Modi cultivars. According to S. Longhi et al. (2013b), cultivar Granny Smith has an allele of 292 bp in homozygote for the DNA marker of the Md-PG1 gene. A similar allelic variant was previously identified in the Modi variety (Longhi et al., 2013a). Accord- ing to the DNA marker MdExp7SSR for the Granny Smith cultivar, the presence of an allelic variant 198:202 bp is known (Costa et al., 2008), which was also confirmed in our study.

Among the cultivars that were used as parental forms for the production of hybrid plants, the genotypes with the most breedingvaluable combinations of allelic variants of two genes simultaneously are the Smeralda and Granny Smith cultivars. According to the MdPG1 10kd marker, the least valu- able allele is 298 bp; it was identified in the Renoir cultivar – 289:298 and in Renet Simirenko – 298:298. At the same time, according to the Md-Exp7 gene marker, an allelic variant was identified in them, which is valuable for selection 202:202, which can probably compensate for the negative effect of al- lelic variants for the Md-PG1 gene. This is supported by the fact that the Renet Simirenko variety, although inferior to the Granny Smith variety in terms of storability, however, exhibits a fairly high level of this trait. At the same time, it is characte rized by a sharp decrease in the density of the fruit flesh with an increase in storage temperature, which cannot be said about the Granny Smith variety, which is the variety with the highest fruit keeping quality (Prichko, 2018; Prichko et al., 2019). It can be assumed that in this way the Renet Simirenko variety showed a negative effect of the 298:298 allelic variant by the DNA marker of the Md-PG1 gene, because, as mentioned above, this gene has a more pronounced contribution to the phenotypic variation in the change in flesh firmness during storage of fruits at temperatures close to room temperature (Costa et al., 2010). In general, the availability of information about allelic combinations of DNA markers of target genes makes it possible to correct pairs of crosses to increase the yield of hybrids with the most valuable allelic combinations.

Considering the distribution of alleles of the DNA marker of the Md-Exp7 gene, we can note hybrid progenies No. 2 and 3, in which all hybrid accessions are homozygous for the 202 bp allele, which corresponds to the allelic variants of the parent varieties (202:202 in all parental forms in these combinations). In hybrid combination No. 5, for which nine plants were analyzed, allelic variants 198:202 and 202:202 were identified, which corresponds to the alleles of the pa- rental varieties. A small sample size does not allow to reliably estimate the deviation of the distribution from the expected 1:1 – (198:202) : (202:202). Specific distribution was observed in progenies No. 1, 4 and 6. Plants with the 214 bp allele predominate in these hybrid populations (allelic variants 202:214 and 198:214) (Table 2). However, taking into account the alleles for the DNA marker of this gene in parental varie ties, the ratio of plants carrying the 202:214 allele variant to plants with the 202 allele in the homozygote (i. e. 202:202) in hybrid combinations No. 1 and 4 should be close to a 1:1 distribution, and in the sample of plants obtained in combi- nation No. 6, the expected distribution is 1:1:1:1 for allelic combinations 198:202, 198:214, 202:202, 202:214. Obvious ly, there is a significant predominance of plants carrying the 214 bp allele

**Table 2.**
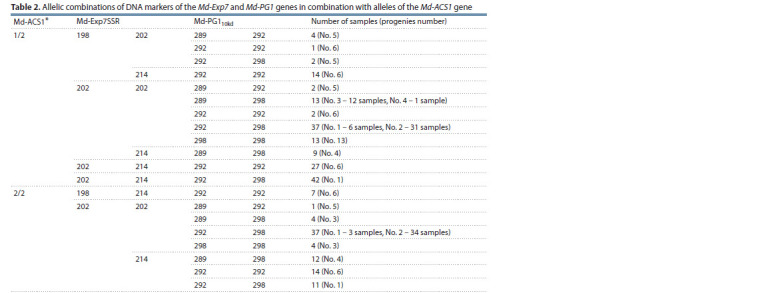
Allelic combinations of DNA markers of the Md-Exp7 and Md-PG1 genes in combination with alleles of the Md-ACS1 gene Allelic variants of the Md-ACS1 gene are indicated, according to the numbering proposed by Sunako et al. (1999)

The reason for the deviation in the distribution of allelic variants is the fact that the Md-Exp7 gene and the Rvi6 scab resistance gene are located on the first chromosome, while the distance between them is about 9 cM (Costa et al., 2008). In this study, mapping of the Md-Exp7 gene was carried out using a hybrid population obtained in a combination of cross- ing varieties Prima (202:214), Rvi6rvi6/Fiesta (202:202), rvi6/ rvi6, which made it possible to establish the distance between these genes.

In our study, in hybrid combinations No. 1, 4 and 6, the Modi variety with an allelic combination of 202:214 bp by DNA marker MdExp7SSR was used as a donor of the scab resistance gene. Taking into account the fact that in the presented work, the analysis of plants carrying the dominant allele of the Rvi6 gene was carried out, we can speak about the regularity of the result obtained and the confirmation of the genetic distance between the Md-Exp7 and Rvi6 genes. The summation of all plants from three hybrid combinations No. 1, 4 and 6 shows that out of 149 plants, an allele of 214 bp is present in 136 plants, and the total number of plants without it is 13 (about 9 % of the total number of plants), which is consistent with the genetic distance between the Md-Exp7 and Rvi6 genes.

For additional verification of the absence of erroneously interpreted results, on the example of the largest hybrid family of the three for which there was a deviation of the observed distribution of alleles from the expected – hybrid family No. 1, a molecular genetic analysis was performed using the MdExp7SSR DNA marker for all hybrid plants, regardless from the presence of a dominant allele of the Rvi6 gene. It was found that out of 231 hybrid plants, 113 have the 202:214 allelic variant, and 118 plants have the 202:202 allelic vari- ant. Thus, there is no significant deviation from the 1:1 ratio (χ2 (1:1) = 0.11 at χ2 crit = 3.8).

The distribution of alleles for the DNA marker of the Md-PG1 gene corresponds to allelic variants in parental cul- tivars: in combinations No. 1, 2, 4 and 6, the hybrid progeny is uniform and has allelic variants 292:298, 292:298, 289:298 and 292:292, respectively. In hybrid combinations No. 3 and 5, two types of allelic combinations are present, consistent with the allelic variants of the parent varieties

**Fig. 2.**
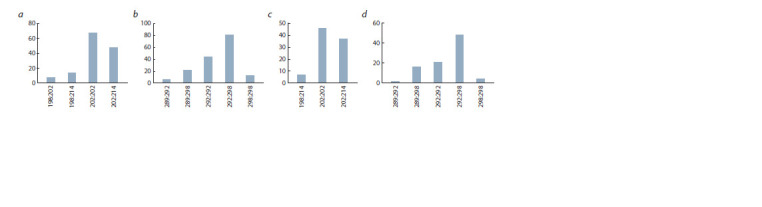
The correlation of allelic variants of the Md-Exp7 (a, c) and Md-PG1 (b, d ) genes in hybrid plants with different allelic variants of the Md-ACS1 gene: 1/2 (a, b) and 2/2 (c, d)

Considering combinations of allelic variants of the Md-Exp7 and Md-PG1 genes with alleles of the Md-ACS1 gene present in the studied apple hybrid accessions, the predominance of the 202:202 bp allelic variant is seen by the DNA marker of the Md-Exp7 gene and 292:298 by the DNA marker of the Md-PG1 gene both in the sample of hybrids with the 1/2 allele variant and in the sample of homozygous for the allele 2 of the Md-ACS1 gene hybrid samples (Fig. 2).

It is also necessary to note a rather high share of plants with an allele set of 202:214 for the DNA marker of the Md- Exp7 gene and 292:292 for the DNA marker of the Md-PG1 gene.

The samples carrying the combination Md-Exp7 (202:202) + Md-ACS1 (2/2) have the highest value for breeding. 46 such accessions were identified. Accessions carrying combinations of alleles Md-PG1 (292:292) + Md-ACS1 (2/2) are also the most valuable for use in breeding and as donors of selection valuable alleles – 21 accessions were identified.

However, given the fact that no homozygous samples for the 214 allele of the Md-Exp7 gene marker were found, and for samples with the allele variant 298:298 (the least priority for selection) for the DNA marker of the Md-PG1 gene, an insignificant number was detected – 17 samples out of 256 of plants included in the study sample, we can talk about the presence of a wide list of breeding forms that are valuable both for further breeding and for use as donors of scab resistance (Rvi6 gene) and a complex of breedingvaluable alleles of several genes simultaneously that determine flesh density – Md-Exp7, Md-PG1 and Md-ACS1. This is supported by the fact that among modern industrial cultivars that are actively used in world horticulture, allele variants that determine the average level of phenotypic expression of the target trait are quite widespread (Costa et al., 2008, 2010; Nybom et al., 2012; Longhi et al., 2013a, b), which is apparently due to the polygenic control of the trait, in which the presence of alleles “average” in terms of selection value simultaneously at the loci of several genes gives the desired phenotypic effect

## Conclusion

Thus, the performed study made it possible to identify groups of apple breeding selections with different combinations of alleles of the Md-Exp7 and Md-PG1 genes among acces- sions carrying the Rvi6 scab resistance gene and possessing selection-valuable allelic variants of the Md-ACS1 gene. The information obtained made it possible to identify donors with a complex of priority alleles that are of high value for use in breeding in order to create new generation varieties that are resistant to apple scab and have a high level of fruit storability.

## Conflict of interest

The authors declare no conflict of interest.
